# L’évolution à long terme du reflux vésico-rénal chez l’enfant

**DOI:** 10.11604/pamj.2019.33.304.18966

**Published:** 2019-08-19

**Authors:** Mohamed Amine Oukhouya, Saad Andaloussi, Mohammed Tazi, Abdelhalim Mahmoudi, Khalid Khattala, Youssef Bouabdallah

**Affiliations:** 1 Service de Chirurgie Pédiatrique, CHU Hassan II, Faculté de Médecine et de Pharmacie, Fès, Maroc

**Keywords:** Reflux vésico-rénal, infection urinaire, enfant, Vesicorenal reflux, urinary tract infection, child

## Abstract

Le reflux vésico-rénal (RVR) est une pathologie assez fréquente chez l'enfant nécessitant un suivi long pour réduire l'évolution vers l'insuffisance rénale chronique. L'objectif de notre étude est d'analyser les aspects épidémiologiques, le diagnostic, la prise en charge du reflux vésico-rénal et l'évolution à long de terme de cette pathologie. Pour cela on a mené une étude rétrospective portant sur une série de 42 patients hospitalisés au Service de Chirurgie Pédiatrique Viscérale du CHU Hassan II de Fès suspectés porteurs d'un reflux vésico-rénal (RVR) sur une période de 6 ans allant de janvier 2010 à décembre 2015. On a trouvé que l'âge médian au moment du diagnostic était de 3 ans et 2 mois. Le sex ratio était de 1,8 garçon pour fille. Le RVR était retrouvé isolé chez 81%, et secondaire ou associé chez 19%. Le principal mode de révélation était l'infection urinaire (90,4%). La fonction rénale était altérée chez 54,8% des enfants. Le traitement a consisté en une antibiothérapie des IU documentées (90,4%) et une antibio-prophylaxie si récidive, une réimplantation de type COHEN dans 97,62%. L'indication chirurgicale a concerné d'emblée tous les grades IV et V (73,9%), ainsi que les reins altérés, et 26,1% après traitement médical. L'évolution postopératoire précoce et tardive était en général satisfaisante: on a noté 92,68% de disparition du RVR. L'UHN a régressé sauf chez 9,52% patients, 95,23% malades ont normalisé leur fonction rénale. La récurrence des IU a diminué (19,04 % après la chirurgie). La majorité des parents ont jugé positivement l'évolution de l'état général de leurs enfants (54,76 %) après la chirurgie.

## Abstract (en)

Vesicoureteral reflux is very common in children, requiring a long follow-up period to reduce its progression toward chronic renal failure. This study aims to analyze the epidemiological diagnostic features, the management of vesicoureteral reflux and long term course of patients with this disease. We conducted a retrospective study of 42 patients with suspected vesicoureteral reflux hospitalized in the Department of Visceral Paediatric Surgery at the Hassan II University Hospital of Fez over a period of 6 years from January 2010 to December 2015. Mean age of patients at diagnosis was 3 years and 2 months. The boy is to girl sex ratio was 1.8. Vesicoureteral reflux was isolated in 81% of patients and secondary or associated in 19% of patients. The most common initial manifestation was urinary tract infection (90.4%). Renal function was impaired in 54.8% of children. The treatment was based on antibiotics against diagnosed UTI (90.4%), antibiotic prophylaxis in the case of recurrence and Cohen reimplantation (97.62%). Surgical indication readily concerned all patients with grades IV and V vesicoureteral reflux (73.9%) as well as patients with impaired kidneys and 26.1% of patients after medical treatment. Early and late postoperative course was in general satisfactory: disappearance of vesicoureteral reflux in 92.68% of cases. Upper urinary tract regressed except for 9.52% of patients; 95.23% of patients had complete recovery of renal function. There was a reduction in UTI recurrence (in 19.04% of cases after surgery). The majority of parents judged positively the clinical course of their children (54.76%) after surgery.

## Introduction

Le reflux vésico-rénal est une uropathie malformative fréquemment rencontrée chez l'enfant qui se définit par l'intrusion d'urine vésicale au niveau du haut appareil urinaire par défaillance de la jonction urétéro-vésicale [1], c'est un phénomène dynamique pouvant être permanant ou intermittent ainsi de sévérité variable chez un même individu. Cependant, sa prévalence dans la population générale est difficile à évaluer car le dépistage de masse chez des sujets sains est éthiquement impossible à concevoir devant les moyens invasifs nécessaires au diagnostic. Qu'il soit primitif ou secondaire, le diagnostic du RVR est souvent posé par la cystographie rétrograde, le plus souvent au décours d'une infection urinaire fébrile, parfois lors d'une anomalie échographique rénale. La guérison spontanée est possible dans 50% à 80% des cas surtout pour les grades I à III [2], mais des complications peuvent survenir, dominées par la néphropathie du reflux: l'hypertension artérielle et le développent d'une insuffisance rénale chronique et terminale. Les deux alternatives thérapeutiques sont : le traitement médical par les antiseptiques urinaires et le traitement chirurgical avec réimplantation de l'uretère ou implant sous muqueux par la voie endoscopique dont l'indication reste difficile en sachant qu'il n'existe jusqu'à ce jour aucun consensus concernant l'approche thérapeutique du RVR. Notre objectif est d'étudier chez une série de 42 patients colligés au Service de Chirurgie Pédiatrique I du CHU Hassan II de Fès entre 2010 et 2015; les aspects épidémiologiques, les conduites diagnostique et thérapeutique du RVR ainsi que son évolution à long terme.

## Méthodes

Il s'agit d'une étude rétrospective portant sur une série de 42 patients hospitalisés au Service de Chirurgie Pédiatrique Viscérale du CHU Hassan II de Fès suspectés porteurs d'un reflux vésico-rénal sur une période de 6 ans allant de janvier 2010 à décembre 2015. L'étude a concerné tous les patients admis au service de chirurgie pédiatrique pour reflux vésico-rénal, dont le diagnostic a été suspecté par l'histoire et le tableau clinique et confirmé radiologiquement, ils ont bénéficié d'un traitement médical et/ou chirurgical. Afin de réaliser cette étude, la collecte des renseignements a été réalisée selon les étapes suivantes: 1) recherche de dossiers des patients ayant été admis pour une suspicion du reflux vésico-rénal à partir des registres du service de chirurgie pédiatrique viscérale (entre 2010 et 2015); 2) traitement des dossiers médicaux et les comptes rendus opératoires puis exclusion de tous les cas ne répondant pas aux critères d'inclusion de notre travail; 3) établissement pour chaque malade, d'une fiche d'exploitation permettant de collecter le maximum de données cliniques et para-cliniques; 4) un questionnaire adapté au niveau intellectuel des parents afin d'étudier l'évolution de l'état sanitaire des malades à long terme rempli par contact téléphonique.

## Résultats

Dans notre étude, l'âge des enfants atteints du RVR a varié entre 2 mois et 15 ans, avec une médiane de 3 ans et 2 mois. Dans notre série, 27 patients sont des garçons (68,28 %) et 15 sont des filles (31,72 %). Une véritable prédominance masculine est constatée, avec un sex-ratio de 1,8. Douze enfants ont une notion de consanguinité parentale: de 1^er^ degré chez 9 enfants et de 2^ème^ degré chez 3 cas. **Diagnostic anténatal**: la découverte sur une échographie faite en anténatal d'une dilatation des cavités excrétrices suspectant un RVR au cours de la croissance est retrouvée dans un seul cas soit 2,4 %. Nous avons noté la présence d'un RVR chez les familles dans 4 cas soit 9,5 % dont un frère, deux sœurs et une mère. Pour le motif de consultation l'infection urinaire vient au premier plan: chez 38 patients soit 90,48%, soit comme premier épisode (chez 11 cas soit 28,95%) soit après plusieurs épisodes (chez 27 cas soit 71,05%). En second plan, viennent les troubles digestifs (4,8 %): la diarrhée et les vomissements dans un cas et les troubles de transit dans un autre cas. Au troisième plan, l'insuffisance rénale chronique terminale chez 2 patients (4 ,8 %). Dans notre étude, l'examen clinique a révélé des signes généraux avec 73,8 % de fièvre, 4,7% d'AEG, 14,2 % de déshydratation puis une distension abdominale sans masse palpable chez 8 cas (19%), et une malformation ano-rectale dans un seul cas (2,4 %). La fonction rénale a été évaluée dans notre étude par les dosages sériques de l'urée et la créatinine, interprétés en fonction de l'âge et du sexe. A l'admission au service, 23 malades avaient une insuffisance rénale soit 54,8 % (dont 2 avaient une IRC suivis en pédiatrie) et 19 malades avec un taux normal. Les taux de l'urée sérique ont varié entre 0,16 g/l et 3,7 g/l et ceux de la créatinine ont varié entre 4,2 et 43 mg/l. Sur 42 ECBU réalisés en préopératoire chez les patients de notre série, 4 sont négatifs (soit 9,5 %), la culture est positive chez 27 malades (64,28%) et une hyperleucocytose à culture négative dans 11 cas (26,2%). Tous les patients de notre série ont bénéficié d'une échographie rénale et vésicale en préopératoire, dont 4 ont été normales (9,5%), L'UHN est le signe le plus fréquent chez nos malades (66,36 %) et souvent bilatérale: 22 cas (52,4 %), unilatérale droite chez 2 patients (4,7 %) et unilatérale gauche chez 4 patients (9,52 %).

**Figure 1:**
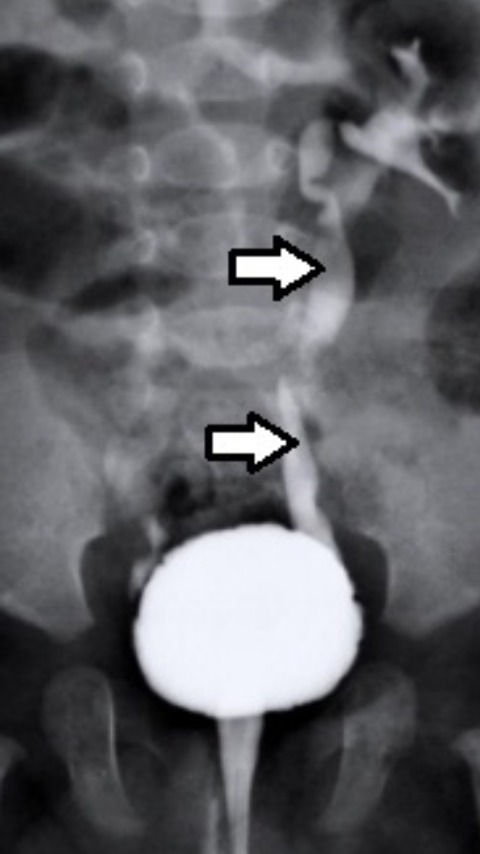
Urétro-cystographie rénale montrant un reflux vésico-rénal droit grade V chez un malade âgé de 3 ans

Tous nos patients ont bénéficié d'une UCGR préopératoire avec une légère prédominance du RVR unilatéral droit, avec 73,9% de haut grade (IV; V) contre 26,1% de faible grade (II; III), alors qu'on n'a noté aucun cas du RVR de grade I (Figure 1). La scintigraphie statique au DMSA a été réalisée chez 21 malades, objectivant : des cicatrices rénales chez 8 cas (3,1%), une asymétrie fonctionnelle entre les deux reins chez 11 cas (52,38%) et un seul cas de rein non fonctionnel (4,7%). Dans notre série, le traitement antibio-prophylactique s'est fait à base de : cotrimoxazole (surlfaméthaxazole+ triméthoprime), la dose est le quart de la dose curative, dont la durée était entre 3 mois et 2 ans. 41 des patients de notre série ont bénéficié d'une réimplantation urétérovésicale type de COHEN soit 97,62%, contre un seul cas soit 2,38% qui a bénéficié une réimplantation selon technique de Lich-Gregoir. L'évolution clinique a été jugée bonne en général avec des troubles mictionnels seulement chez 3 patients (Tableau 1), seulement 8 patients ont fait des infections urinaires après l'intervention contre 38 avant l'intervention (Tableau 2 et Tableau 3). Seulement 3 patients de notre série présentaient une persistance radiologique du RVR

**Tableau 1:** Répartition des cas selon la durée du suivi

Auteur	Année	Nombre patients	Recul	Résultats
Bailey	1992	21 enfants	24 ans	Evolution satisfaisante: pas de néphropathie
Beetz	2002	158 enfants	20,3 ans	50% d’IU surtout basses et 17 % de PNA
Notre étude	2017	42 enfants	4,5 ans	Evolution favorable: IU: 19,04%

**Tableau 2:** Récurrence des infections urinaires après traitement du RVR

Auteur	Nombre de cas	Evolution d’IU	Recul
Aubert	109	10% de récidive	4 ans
Hyne	48	25% de récidive	1 an
Rice	45	27% de récidive	1,5 an
Notre série	42	19,04% de récidive	4,5 ans

**Tableau 3:** Comparaison des résultats d'ECBU avant et après traitement

Résultats	ECBU avant le traitement	ECBU après le traitement
ECBU négatif	9,5%	76,19%
ECBU à culture positive	64,28%	19,04%
ECBU avec hyperleucocytose positive et culture négative	26,2%	4,76%

## Discussion

Le reflux vésico-urétéro-rénal est uropathie malformative lourde de conséquences, nécessitant un diagnostic précoce, une prise en charge correcte et adéquate, afin d'éviter la destruction progressive du parenchyme et l'installation d'une insuffisance rénale irréversible. L'avènement de l'échographie anténatale a transformé le pronostic vital et fonctionnel des uropathies congénitales. Le reflux vésico-urétéro-rénal peut être retrouvé lors de l'investigation d'une dilatation des cavités urinaires excrétrices diagnostiquée en anténatale dans 10 à 20% des cas. L'infection urinaire est le mode principal de révélation du RVR chez l'enfant et son mode de présentation varie selon l'âge. L'insuffisance rénale et l'hypertension artérielle sont plutôt des complications redoutables des uropathies malformatives et c'est exceptionnellement qu'elles constituent une cause révélatrice [3, 4]. L'échographie n'est pas l'examen de référence pour le diagnostic d'un RVR, car 25% des dilatations anténatales secondaires à un RVR, et qui ont été confirmées en postnatale avaient disparu [5].L' UCGR est devenue l'examen de référence pour poser le diagnostic du RVR à cause de sa spécificité et l'analyse anatomique qu'elle permet du haut et du bas appareil urinaire. Cependant sa sensibilité diagnostic reste faible puisque un RVR est retrouvé secondairement dans 20% des UCGR initialement normales [6].cette même étude avait confirmé que le RVR est un phénomène dynamique, de sévérité variable dans le temps chez un même individu.

**La scintigraphie statique au DMSA** visualise les cicatrices rénales focales et parfois détecte l'atteinte parenchymateuse dans la pyélonéphrite aiguë et les cicatrices rénales ou, plus tardivement, la présence de séquelles cicatricielles, et la quantification de la filtration rénale séparée [7]. Le traitement médical consiste en une antibiothérapie prophylactique, utilisant des antibiotiques à doses sub-inhibitrices (le quart de la dose efficace), poursuivi tant que persiste un reflux significatif avec un traitement dans les premiers mois qui est un impératif [8]. Deux types de traitement chirurgical du RVR sont possibles : le traitement endoscopique et la réimplantation urétéro-vésicale qui tous deux, visent à allonger le trajet sous-muqueux de l'uretère selon plusieurs techniques. Le suivi repose essentiellement sur l'évaluation clinique et la récurrence des infections urinaires, l'évaluation biologique en guettant l'insuffisance rénale, et l'évaluation radiologique.

## Conclusion

Au terme de notre travail, le suivi de 42 cas nous ramène à tirer certaines conclusions: le reflux vésico-rénal est une uropathie malformative fréquente chez l'enfant dont le but de sa prise en charge est de prévenir l'apparition ou l'aggravation de la néphropathie du reflux: HTA et l'insuffisance rénale chronique et terminale. Le tableau clinique est polymorphe, dominé par des infections urinaires à répétition. Les examens radiologiques visent à confirmer l'infection urinaire et biologique à apprécier le retentissement du reflux vésico-rénal sur le parenchyme rénal. L'urétro-cystographie rénale reste l'examen clé pour poser le diagnostic du reflux vésico-rénal. Le traitement chirurgical est indiqué devant un RVR responsable d'infections urinaires fébriles, malgré une prévention antibiotique correcte et une prise en charge des troubles mictionnels. En l'absence de ces signes, le traitement médical conservateur est tenté. L'évolution des patients opérés pour un RVR est favorable, malgré la persistance de l'infection fébrile dans 19,04 % des cas. La tolérance à long terme d'une réimplantation urétérale est satisfaisante; jugée sur: 1) la disparition ou la régression des épisodes d'infections urinaires; 2) la normalisation ou l'amélioration de la fonction rénale; 3) la disparition des anomalies radiologiques notamment l'UHN à l'échographie de contrôle; 4) la régression des lésions rénales à la scintigraphie rénale.

### Etat des connaissances actuelles sur le sujet

- Le reflux vésico-rénal est une uropathie fréquente chez l'enfant;
- Les résultats satisfaisants de la technique de réimplantation de Cohen chez la population pédiatrique.

### Contribution de notre étude à la connaissance

- Les résultats à long terme favorables de la réimplantation chirurgicale;
- Evaluer le retentissement du retard de la prise en charge sur l'évolution.

## Conflits des intérêts

Les auteurs ne déclarent aucun conflit d’intérêts.
